# Unilateral ovarian recurrence 5 years after hysterectomy for microinvasive squamous cervical cancer stage IA1: a rare case report

**DOI:** 10.1186/s12905-023-02496-9

**Published:** 2023-07-01

**Authors:** Longxia Tong, Lin Wu

**Affiliations:** 1 Department of Obstetrics and Gynecology, West China Second University Hospital, Sichuan University, Chengdu, 610041 China; 2 Key Laboratory of Birth Defects and Related Diseases of Women and Children (Sichuan University), Ministry of Education, Sichuan Province, No. 20, 3Rd Section, South Renmin Road, Chengdu, 610041 China

**Keywords:** Squamous cell carcinoma, Microinvasive, Cervical cancer, Ovarian recurrence, Case report

## Abstract

**Background:**

Ovarian metastasis or recurrence of cervical microinvasive squamous cell carcinoma (SCC) is very rare. We report a case of unilateral ovarian recurrence 5 years after hysterectomy for the SCC stage IA1 without lymph vascular space invasion (LVSI).

**Case presentation:**

A 49-year-old female patient suffered from a dull pain in the left lower abdomen for 3 months. And five years ago, she received a laparoscopic hysterectomy for the treatment of stage IA1 (without LVSI) SCC of the cervix. The level of squamous cell carcinoma antigen (SCC-Ag) in serum was significantly elevated (10.60 ng/mL). Pelvic magnetic resonance imaging (MRI) revealed a left ovarian solid tumor measuring 5.5 × 3.9 × 5.6 cm with heterogeneous enhancement. During laparotomy, the left ovarian tumor was measured about 5.0 × 4.5 × 3.0 cm and seemed densely adherent to the posterior peritoneal wall, including the left ureter. The tumor and pelvic lymph node were carefully removed. Postoperative anatomy revealed a solid mass with a greyish-white section. Postoperative pathology showed recurrent moderately differentiated ovarian SCC with negative pelvic lymph nodes. Immunohistochemistry showed that the tumor cells were positive for P16, P63, P40, and CK5/6 markers, and the positive rate of Ki67 was about 80%.

**Conclusions:**

Ovary preservation is reasonable and appropriate in young patients with microinvasive SCC. Ovarian recurrence is rare, but gynecological oncologists should not overlook its possibility. The serum SCC-Ag is an important indicator for monitoring postoperative disease progression.

## Background

Previous studies have shown a good prognosis for microinvasive squamous cervical cancer [1]. The standard treatment the National Comprehensive Cancer Network recommends is a simple hysterectomy or cervical conization for patients with microinvasive squamous cervical cancer without LVSI if there is no fertility requirement. The ovarian metastasis (OM) incidence is relatively low in patients with squamous cell carcinoma (SCC). Ovarian preservation can be safely performed in young patients [2]. Primary ovarian SCC is extremely rare. Most cases are caused by cystic teratoma malignancy, and very few cases are caused by Brenner tumor or endometriosis. A review of the published literature revealed only one case of ovarian SCC recurred 8 years after fertility-conserving surgery for early microinvasive SCC [3]. This was the second case of unilateral ovarian recurrence 5 years after a hysterectomy for microinvasive squamous cervical cancer stage IA1.

## Case presentation

**Fig. 1:**
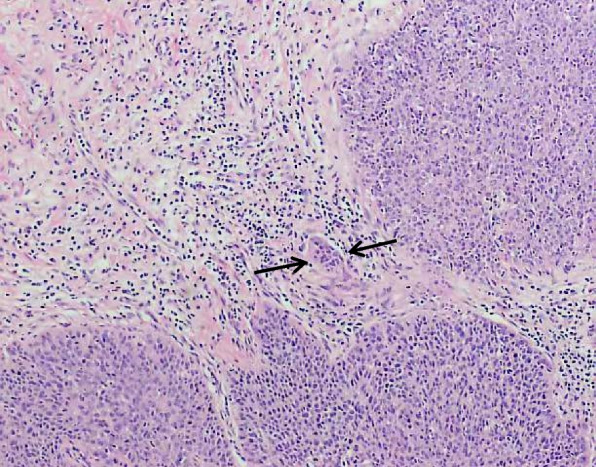
Microinvasive squamous cell carcinoma (SCC), with a maximal depth of 1 mm (arrow pointing to focus of microinvasive front line) (hematoxylin–eosin stain, original magnification, × 400)

A 49-year-old female patient suffered from a 3-month history of dull pain in the left lower abdomen. And she had laparoscopic hysterectomy for the microinvasive SCC stage IA1 in 2018. Five years ago, the patient was admitted to our hospital with postcoital vaginal bleeding. The cervical biopsy pathology indicated grade III cervical intraepithelial neoplasia (CIN III) with the involvement of glands and human papillomavirus (HPV) type 16 DNA positive. The cervical conical resection was performed. The postoperative examination indicated extensive CIN III with glandular involvement. The microscopic invasive SCC (both width and depth less than 1 mm) was found in the focal area, without lymph vascular space invasion (LVSI) (Fig. 1). Because the patient had no fertility requirement, laparoscopic total hysterectomy and bilateral salpingectomy were performed, and no more severe lesions were found on the postoperative pathological examination.

**Fig. 2:**
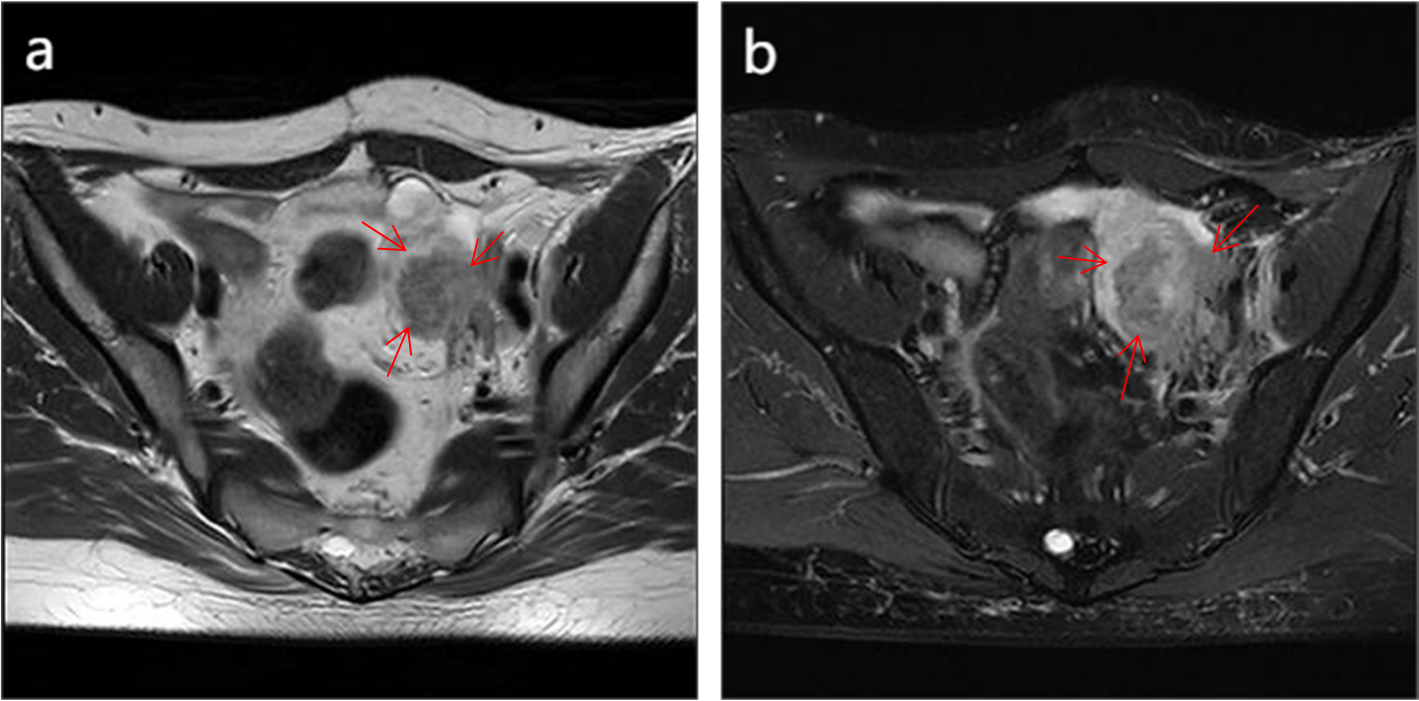
Pelvic magnetic resonance imaging demonstrating a pelvic solid tumor with marked enhancement. On T1-weighted magnetic resonance imaging, the tumor showed hypointensity. After administration of gadolinium-DTPA, the delayed phased image showed heterogenous tumor enhancement (arrow indicates pelvic tumor)

**Fig. 3:**
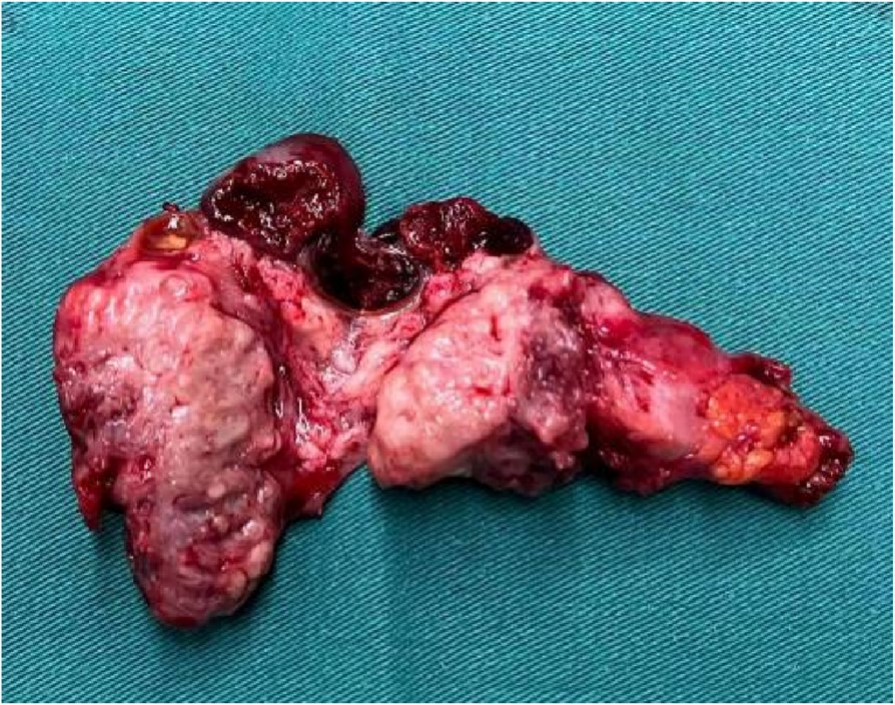
Postoperative anatomy showed a solid mass with a greyish-white section measuring about 5.0 × 4.5 × 3.0 cm.^3^

**Fig. 4:**
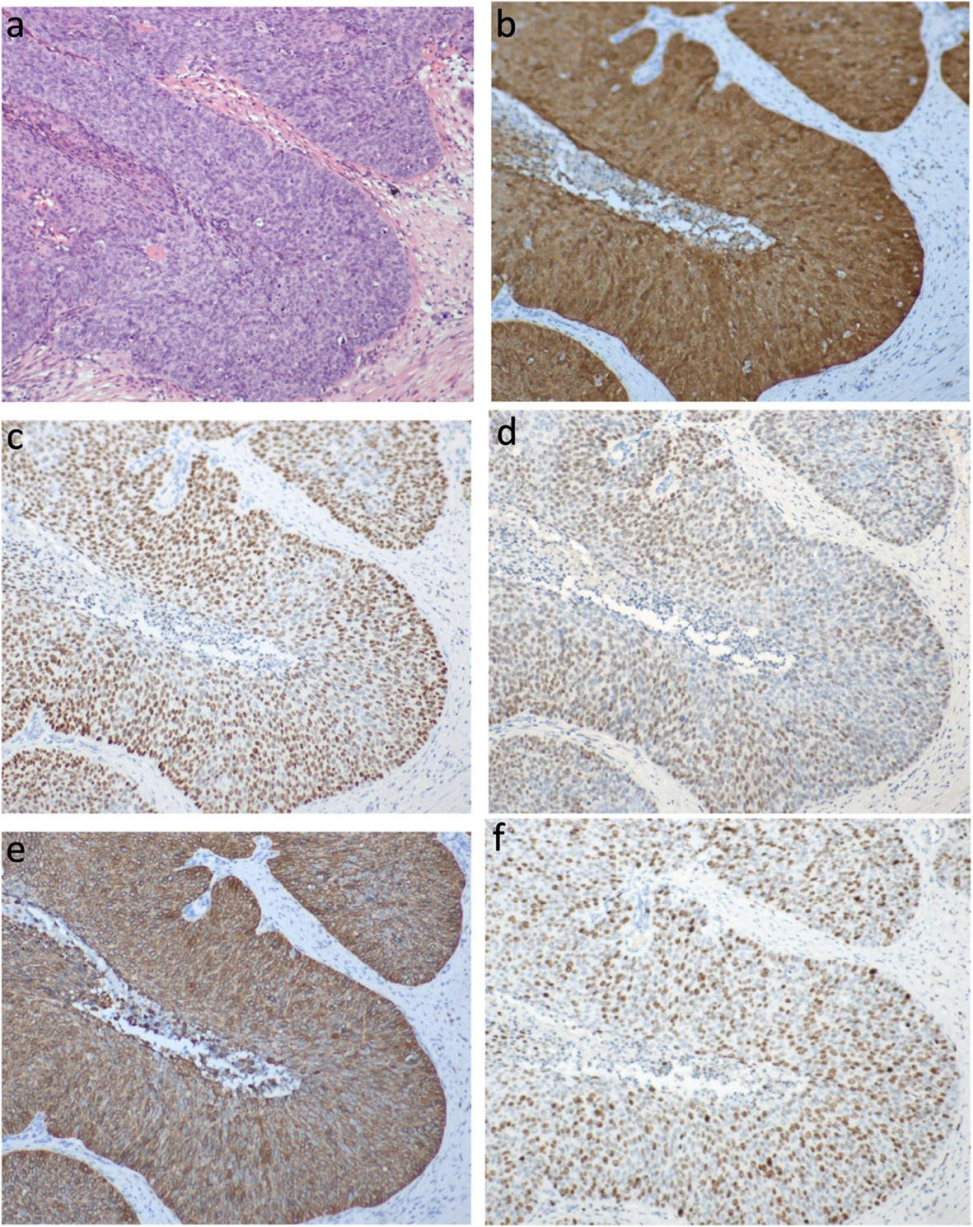
**a** Medium-grade SCC (hematoxylin–eosin stain,original magnification, × 100); immunostaining of the ovarian tumor **b** Ovarian tumor cells were positive for the p16 marker (original magnification, × 100), **c** Cells positive for the P63 marker (original magnification, × 100), **d** Cells positive for the P40 marker (original magnification, × 100), **e** Cells positive for the CK5/6 marker (original magnification, × 100), **f** The positive rate of Ki67 was about 80% (original magnification, × 100)

The patient was followed up regularly after surgery, and no abnormalities were found in cervical cytology, HPV infection, and squamous cell carcinoma antigen (SCC-Ag) during an annual review. Five years after surgery, she complained of a 3-month history of dull pain in the left lower abdomen. Physical examination showed a palpable mass on the left side of the pelvic cavity, and examination of the vulva, vagina, and vaginal stump was normal. The cytology and HPV test results of vaginal stump exfoliation were negative. The levels of the tumor marker of SCC-Ag in serum showed marked elevation (10.60 ng/mL), and the carbohydrate antigen 125 was normal. The sonographic examination demonstrated a left ovarian solid mass measuring 4.2 × 3.4 × 3.6 cm. Pelvic MRI demonstrated a left ovarian solid tumor measuring 5.5 × 3.9 × 5.6 cm with heterogeneous enhancement (Fig. 2a and b), and increased left pelvic lymph nodes were also demonstrated. The positron emission tomography-computed tomography (PET-CT) did not reveal the possibility of metastatic tumors or tumors derived from other origins. Therefore, an exploratory laparotomy was performed. During laparotomy, a left ovarian tumor measuring about 5.0 × 4.5 × 3.0 cm seemed densely adherent to the posterior peritoneal wall, including the left ureter, which was resected completely. Pelvic lymph nodes were suboptimal resection due to the tumor adhering closely to the pelvic sidewall. Postoperative anatomy revealed a solid mass with a greyish-white section (Fig. 3). The right ovarian was resected, and no abnormal appearance was observed. Postoperative pathology showed moderately differentiated SCC with negative pelvic lymph nodes (Fig. 4a). Immunohistochemistry showed that the tumor cells were positive for P16, P63, P40, and CK5/6 markers, and the positive rate of Ki67 was about 80% (Fig. 4b-f). No endometriosis or teratoma components were found. The possibility of primary ovarian SCC could not be completely ruled out; however, the ovarian tumor was considered to be derived from the microinvasive SCC of the cervix stage IA1 in combination with the patient's medical history and immunohistochemical examination. Following surgery, the serum SCC-Ag decreased to 3.8 ng/mL on postoperative day 7. The patient was supplemented with radiotherapy and chemotherapy after surgery, there was no sign of tumor recurrence, and long-term follow up will be conducted to observe the prognosis.

## Discussion and conclusions

The OM of cervical cancer is rare. A recent systematic review showed that the incidence of OM in stage IA to IIB cervical carcinoma was 1.46% [4]. For patients with microinvasive squamous cervical cancer without LVSI, the standard treatment is a simple hysterectomy or cervical conization if there is no fertility requirement. Ovary preservation is reasonable and appropriate in young patients because oophorectomy seriously affects their long-term quality of life and increases the risk of osteoporosis and cardiovascular diseases.

The recurrence rates of different stages of cervical cancer were about 10% for FIGO Stage IB, 17% for IIA stage, 23% for IIB stage, 42% for III stage and 74% for IVA stage [5]. The recurrence rate for tumors ≤ 2 cm has an estimated recurrence risk of only 1.2% while for tumors larger than 2 cm the recurrence rate is as high as 21% [6, 7]. The symptoms of cervical cancer recurrence are usually insidious, including recurrence of local pelvic disease, para-aortic lymph nodes, and distant recurrence. Previous studies reported that distant-only (DO) recurrence was most common (59.5%), followed by combined (21.5%), central (cervix or vaginal stump;10.7%), and pelvic (pelvic lymph nodes or pelvic side wall; 8.3%) recurrence [8]. There has been considerable controversy regarding ovarian preservation in patients with cervical adenocarcinoma. Current studies have shown that ovarian preservation in young women with early-stage cervical AC might be safe [9]. Ovarian recurrence is extremely rare for microinvasive squamous cervical cancer. A review of the published literature revealed only one case of ovarian SCC recurred 8 years after fertility-conserving surgery for early microinvasive SCC with positive lymphovascular involvement [3]; this was the second case of unilateral ovarian recurrence 5 years after hysterectomy for the microinvasive squamous cervical cancer stage IA1 without LVSI.

Follow-up visits should include a complete physical examination, history, and pelvic rectal examination, and necessary laboratory and imaging tests as indicated by clinical symptoms. The serum SCC-Ag is consistently associated with the recurrence and mortality of newly diagnosed cervical cancer, and this marker can be used to monitor disease progression in patients with cervical cancer [10, 11]. The patient's treatment history, recurrence/metastasis site, and lesion degree should be comprehensively evaluated, combined with the general condition of the patient. The organic combination of multiple treatment modes, such as radical radiotherapy, pelvic dissection, palliative chemotherapy, and targeted therapy, should be given selectively. Patients with metastatic recurrent cervical cancer have a poor prognosis despite salvage therapy. The patient in the case reports of Takao underwent debulking surgery and was treated with adjuvant chemotherapy consisting of paclitaxel and carboplatin, followed by radiotherapy, but the patient showed progressive disease [3].

In conclusion, we reported a case of ovarian recurrence 5 years after hysterectomy for the microinvasive SCC stage IA1 without LVSI. Our findings suggested that although ovarian recurrence for the microinvasive SCC stage IA1 was rare, gynecological oncologists should not overlook its possibility.

## Data Availability

The data that support the findings of this study are available on reasonable request from the corresponding author. The data are not publicly available due to privacy or ethical restrictions.

## Abbreviations

SCC: Squamous cell carcinoma
LVSI: Lymph vascular space invasion
SCC-Ag: Squamous cell carcinoma antigen
MRI: Magnetic resonance imaging
OM: Ovarian metastasis
CIN III: Cervical intraepithelial neoplasia
HPV: Human papillomavirus

## References

[1] Hartman CA, Teixeira JC, Barbosa SB, Figueiredo SM, Andrade LA, Bastos JF (2017). Analysis of Conservative Surgical Treatment and Prognosis of Microinvasive Squamous Cell Carcinoma of the Cervix Stage IA1: Results of Follow-Up to 20 Years. Int J Gynecol Cancer.

[2] Tewari KS, Sill MW, Long HJ (2014). Improved survival with bevacizumab in advanced cervical cancer. N Engl J Med.

[3] Hidaka T, Nakashima A, Hasegawa T, Nomoto K, Ishizawa S, Tsuneyama K, Takano Y, Saito S (2011). Ovarian Squamous Cell Carcinoma Which Metastasized 8 Years After Cervical Conization for Early Microinvasive Cervical Cancer: A Case Report. Jpn J Clin Oncol.

[4] Fan Yu, Wang M-Y, Yi Mu, Mo S-P, Zheng Ai, Li J-K (2020). Ovarian metastasis in women with cervical carcinoma in stages IA to IIB A systematic review and meta-analysis. Medicine (Baltimore).

[5] Perez CA, Grigsby PW, Nene SM, Camel HM, Galakatos A, Kao MS, et al. Effect of tumor size on the prognosis of carcinoma of the uterine cervix treated with irradiation alone. Cancer. 1992;69:2796e806. 10.1002/1097-0142(19920601)69:11<2796::aid-cncr2820691127>3.0.co;2-o 1571911

[6] Marchiolé P, Buénerd A, Benchaib M, Nezhat K, Dargent D, Mathevet P. Clinical significance of lympho vascular space involvement and lymph node micrometastases in early-stage cervical cancer: a retrospective case-control surgicopathological study. Gynecol Oncol. 2005;97(3):727e32. 10.1016/j.ygyno.2005.01.004 15943983

[7] Grisaru DA, Covens A, Franssen E, Chapman W, Shaw P, Colgan T, et al. Histopathologic score predicts recurrence free survival after radical surgery in patients with stage IA2-IB1–2 cervical carcinoma. Cancer. 2003;97(8):1904e8. 10.1002/cncr.11269 12673716

[8] Kim TH, Kim MH, Kim BJ, Park SI, Ryu SY, Cho CK (2017). Prognostic Importance of the Site of Recurrence in Patients With Metastatic Recurrent Cervical Cancer. Int J Radiat Oncol Biol Phys.

[9] Should ovaries be removed or not in early-stage cervical adenocarcinoma: a multicenter retrospective study of 105 patients. Hu J, Jiao X, Yang Z, Cui H, Guo H, Wu Y, Zhu L. J Obstet Gynaecol. 2017;37(8):1065–1069. 10.1080/01443615.2017.1323198 28631536

[10] Charakorn C, Thadanipon K, Chaijindaratana S, Rattanasiri S, Numthavaj P, Thakkinstian A (2018). The association between serum squamous cell carcinoma antigen and recurrence and survival of patients with cervical squamous cell carcinoma: A systematic review and meta-analysis. Gynecol Oncol.

[11] Chung Chang,Jiabin Chen,Chi-Hsiang Huang,Wen-Ying Lee,Li-Chuan Hsu,An Jen Chiang.Time-Dependent Squamous Cell Carcinoma Antigen in Prediction of Relapse and Death of Patients With Cervical Cancer. J Low Genit Tract Dis. 2020;24(1):38–42. 10.1097/LGT.0000000000000499 31860573

